# Comparison of ocular higher-order aberrations after SMILE and Wavefront-guided Femtosecond LASIK for myopia

**DOI:** 10.1186/s12886-017-0431-5

**Published:** 2017-04-07

**Authors:** Xiaoqin Chen, Yan Wang, Jiamei Zhang, Shun-nan Yang, Xiaojing Li, Lin Zhang

**Affiliations:** 1 Tianjin Eye Hospital & Eye Institute, Tianjin Key Laboratory of Ophthalmology and Visual Science, Tianjin Medical University, No 4. Gansu Rd, Heping District, Tianjin, 300020 China; 2 Vision Performance Institute, Pacific University College of Optometry, Forest Grove, OR USA; 3 First Hospital of Qinhuangdao, Qinhuangdao, Hebei China

**Keywords:** Wavefront aberration, Small incision lenticule extraction, Wavefront-guided femtosecond LASIK, Visual quality, Refractive surgery

## Abstract

**Background:**

To compare changes in higher-order aberrations (HOAs) following small incision lenticule extraction (SMILE) and wavefront-guided femtosecond laser-assisted in situ keratomileusis (WFG FS-LASIK), and to investigate correlations between preoperative spherical equivalence (SE) and components of HOAs affecting visual quality.

**Methods:**

Sixty-five myopic eyes from 38 patients were enrolled in the study retrospectively, either having undergone SMILE or WFG FS-LASIK. Uncorrected distance visual acuity (UDVA), corrected distance visual acuity (CDVA), refractive error, and individual Zernike coefficients of 3rd- to 6th-order HOAs were measured before and 3 months after the surgeries and were compared using the Mann-Whitney test or Student’s t-test. Additional generalized estimating equation analyses (GEE) were used to control for within-subject biases in individual Zernike coefficients between the right and left eyes of the same patients.

**Results:**

There was no significant difference in UDVA or CDVA after WFG FS-LASIK (Mean ± SD: −0.02 ± 0.07 and −0.04 ± 0.22 respectively, in logMAR) and after SMILE (−0.01 ± 0.06 and −0.04 ± 0.04 respectively). However, greater vertical coma aberration was found after SMILE (*p* = 0.036). Preoperative SE was correlated to induced horizontal coma (*r* = −0.608, *p* = 0.001) in WFG FS-LASIK, and correlated to induced vertical coma (*r* = −0.459, *p* = 0.003) in SMILE.

**Conclusions:**

Both SMILE and WFG FS-LASIK can achieve planned visual outcomes in correcting myopia and myopic astigmatism. However, higher vertical coma was shown in SMILE than WFG FS-LASIK which might be a potentially impact factor for patients’ vision under certain lighting conditions and needs further investigation.

## Background

Ocular refractive surgeries require precise corneal correction to achieve ideal visual outcomes and visual quality. Femtosecond laser (FL) utilizes ultrafast pulses to create precise ocular tissue ablation [1], and is commonly used in corneal refractive surgeries to create corneal flaps in femtosecond laser-assisted in situ keratomileusis. Recently, FL has also increasingly been used to create a transparent refractive lenticule in femtosecond lenticule extraction (FLEx) with a lifted corneal flap, and in small-incision lenticule extraction (SMILE) without a lifted flap [2].

SMILE has been considered as an alternative procedure to conventional laser in-situ keratomileusis (LASIK) because of its potential advantages of reduced denervation, faster resolution of post-operative dry eye, improved biomechanics, and no flap-related risks [3–6]. Previous studies showed that increased higher-order aberrations (HOAs) associated with traditional LASIK could cause glare and halos in night vision. Several recent studies have shown that smaller HOAs were induced after SMILE as compared to traditional LASIK [7, 8]. Wavefront-guided LASIK has been shown to correct preexisting aberrations and to result in less postoperative HOAs [9–11]. FL-assisted ablation in wavefront-guided femtosecond LASIK could also potentially reduce induced HOAs [12].

Key procedural differences between SMILE and wavefront-guided femtosecond laser-assisted in situ keratomileusis (WFG FS-LASIK) could contribute to the noted differences in optical qualities following the procedures. SMILE relies on subjective fixation on a target light without eye tracking and iris registration. In contrast, WFG FS-LASIK utilizes iris registration to trace the pupil shift. Pupil shift might affect these two procedures differently, leading to distinct types of HOAs and discordant changes in visual acuity and refraction after SMILE and WFG FS-LASIK.

Therefore, it is necessary to investigate the visual and optical outcomes of WFG FS-LASIK and SMILE and to relate these outcomes to the HOAs induced by the procedures. The present study was the first attempt to determine whether SMILE induces smaller HOAs and achieves better visual outcomes and optical quality than WFG FS-LASIK.

## Methods

### Patients

This study retrospectively evaluated 65 eyes from 38 patients, including 39 eyes from 23 patients in the SMILE group and 26 eyes from 15 patients in the WFG FS-LASIK group. In all of these patients, refractive errors were stable (a change of ±0.50 D or less) for at least 1 year prior to surgery. The corneas of the included eyes were transparent with a central thickness greater than 500 μm and a calculated residual stroma not less than 250 μm. The intraocular pressures (IOPs) of these eyes measured less than 21 mmHg. Patients included in this study did not have any of the following systemic or ocular conditions: diabetes mellitus, connective tissue disease, amblyopia, corneal disease, cataracts, glaucoma, or retinal disease. Patients wearing rigid contact lenses were instructed to stop wearing them at least 4 weeks prior to the surgery, and those wearing soft contact lenses were instructed to stop wearing them at least 2 weeks prior.

All patients underwent comprehensive exams before surgery and again 3 months after surgery. The exams included assessments of uncorrected distance visual acuity (UCVA), corrected distance visual acuity (CDVA), manifest and cycloplegic refraction, pupil size, slit-lamp exam, dilated fundus exam, anterior segment tomography (Pentacam-HR, Oculus GmbH, Wetzlar, Germany), IOP with non-contact tonometry (Topcon-CT80; Topcon, Tokyo, Japan), and measurement of wavefront aberrations (Wavescan; VISX, Santa Clara, CA). Full descriptions of the two surgical procedures were provided to all patients, including the potential advantages, disadvantages, and complications. This study was approved by the Ethics Committee of Tianjin Eye Hospital and adhered to the tenets of the Declaration of Helsinki. Informed consent to use any clinical data for analysis and publication was obtained from all patients prior to surgery.

### Surgical procedures

Both the SMILE and the wavefront-guided femtosecond LASIK procedures were performed by an experienced surgeon (Dr. Yan Wang) at the Tianjin Eye Hospital.

#### SMILE procedure

A detailed description of the SMILE procedure was previously published and is only briefly summarized here [13]. The SMILE surgery was performed using a 500-kHz Visu Max femtosecond laser (Carl Zeiss, Meditec AG, Jena, Germany) with a laser energy of approximately 170 nJ. Following the application of topical anesthesia (oxybuprocaine eye drops, Benoxil, Santen, Inc., Japan), the patient was required to fixate on an internal target light before corneal suction was initiated. The posterior surface of the lenticule was cut first from the periphery to the center, followed by cutting of the anterior surface from the center to the periphery. The diameter of the refractive lenticule measured from 6 to 6.5 mm with a transition zone of 0.1 mm. The incision for lenticule retrieval was made at the 12 o’clock position on the cornea and had a length of 2 to 5 mm with an average of 3.73 mm. The target cap thickness was 110 μm. A manual spatula was inserted through the small incision to dissect the surface plane, and a pair of forceps was used to extract the intrastromal lenticule.

#### Wavefront-guided femtosecond LASIK procedure

WFG FS-LASIK procedures were performed using a VISX STAR S4 excimer laser system (VISX Inc., Santa Clara, USA) after the creation of a 110 μm corneal flap with the femtosecond laser. The temporal-hinged corneal flap was made in the requirement of the Ethics Committee of Tianjin Eye Hospital because studies showed horizontal-hinged flap may cause less loss of sensation and presence of dry eye syndrome than superior-hinged flap in LASIK [14, 15]. Refraction and wavefront information for the treated eyes was collected using a WaveScan system and after being carefully scanned and selected was transferred to the STAR S4 excimer laser system. The operative parameters of the excimer laser were as follows: emission wavelength 193 nm, energy fluence 160 mJ/cm^2^, repetition rate 10 Hz, diameter of ablation area 6.0 to 7.0 mm with transition zone of 0.5 mm. An eye tracker was automatically activated during the laser ablation period. The flap was repositioned then the interface was irrigated with a balanced saline solution.

After both procedures, 0.3% ofloxacin (Tarivid, Santen, Inc., Japan) eye drops were instilled 4 times daily for 3 days and 0.1% fluorometholone (Flumetholon, Santen, Inc., Japan) eye drops were instilled 4 times daily for the first 2 weeks. The 0.1% flurometholone drops were then gradually tapered, reducing the frequency of instillation every 2 weeks (3 times daily, then 2 times daily, and finally once daily). All patients returned for evaluation 3-month postoperatively, no complications were found at this examination.

### Outcome measurements

All HOAs were measured by a WaveScan system using a Hartmann-Shack sensor (Wavescan; VISX, Santa Clara, CA) without pharmacological pupil dilatation following 10 min of dark adaptation. The aberrometer was set at a diameter of 5 mm. The absolute coefficients of oblique trefoil, horizontal trefoil, vertical coma, horizontal coma, and spherical aberration were obtained since the magnitudes of the aberration could reflect the optical quality directly [16]. The levels of HOAs were documented as their root mean square values (RMS, in micrometers).

### Statistical analyses

Measured outcomes were analyzed using the SPSS version 20.0 (IBM Inc., New York, USA). The frequency distribution of preoperative and postoperative outcomes were assessed using the Shapiro-Wilks test. Comparisons between preoperative and postoperative data were performed using the Mann-Whitney test for non-normally distributed data and the Student’s *t*-test for normally distributed data. Additional generalized estimating equation (GEE) analyses were conducted to statistically control the contribution of selected (left versus right) eyes of the same patients in preoperative and postoperative individual Zernike coefficients. To examine the effects of preoperative spherical equivalent (SE) on surgical outcomes, correlations between preoperative SE and the magnitude of individual Zernike coefficients were calculated using the Pearson or Spearman correlation method. All analyses adopted an alpha of 0.05 to determine their statistical significance.

## Results

Patient demographics and preoperative data are summarized in Table 1. There was no significant difference in any of the variables between the SMILE and WFG FS-LASIK groups.

**Table 1:** Demographics and preoperative data of the study population

Mean ± SD (range)	WFG FS-LASIK	SMILE	*P* value ^a^
Number of eyes	26	39	-
Age (years)	24 ± 5	22 ± 4	0.186^b^
Gender (% female)	40%	33%	0.542
UDVA (logMAR)	0.99 ± 0.37 (0.30,1.70)	0.96 ± 0.28 (0.52,1.40)	0.737
CDVA (logMAR)	0.03 ± 0.06 (0.00,0.22)	0.01 ± 0.03 (0.00,0.10)	0.076
Sphere (D)	−4.20 ± 2.65 (−10.00,0.00)	−4.41 ± 1.23 (−6.50,-2.00)	0.710^b^
Cylinder (D)	−2.66 ± 1.12 (−5.50,-1.00)	−2.26 ± 0.73 (−4.00,-1.00)	0.102^b^
SE (D)	-5.54 ± 2.40 (−10.50,-2.00)	−5.53 ± 1.24 (−8.00,-2.88)	0.998^b^
Ocular aberration (μm)			
Z_3_^−3^	0.062 ± 0.063 (0.001,0.211)	0.067 ± 0.047 (0.006,0.206)	0.249
Z_3_^−1^	0.074 ± 0.045 (0.000,0.203)	0.088 ± 0.068 (0.001,0.277)	0.738
Z_3_^1^	0.049 ± 0.043 (0.001,0.168)	0.034 ± 0.029 (0.001,0.126)	0.194
Z_3_^3^	0.040 ± 0.037 (0.000,0.163)	0.043 ± 0.035 (0.000,0.147)	0.698
Z_4_^0^	0.047 ± 0.027 (0.005,0.108)	0.056 ± 0.034 (0.001,0.116)	0.280^b^
Total HOA	0.158 ± 0.055 (0.078,0.281)	0.166 ± 0.063 (0.057,0.370)	0.659

CDVA = corrected distance visual acuity (with spectacles); UDVA = uncorrected distance visual acuity; SE = spherical equivalent; ^a^ *P* values were based on Mann-Whitney test for non-normally distributed data unless otherwise specified; ^b^ *P* values were based on Student t-test for normally distributed data

### Postoperative visual acuity and refraction

**Fig. 1:**
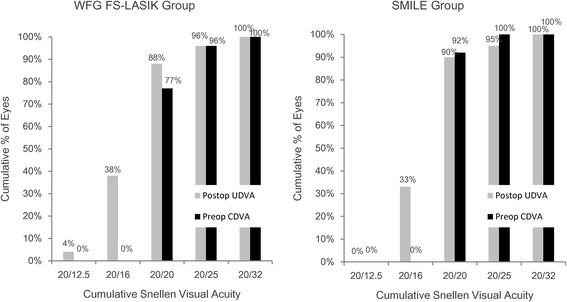
Cumulative percentages of eyes attaining specified cumulative levels of uncorrected distance visual acuity (UDVA) 3 months after wavefront-guided femtosecond LASIK (WFG FS-LASIK) and small incision lenticule extraction (SMILE)

**Fig. 2:**
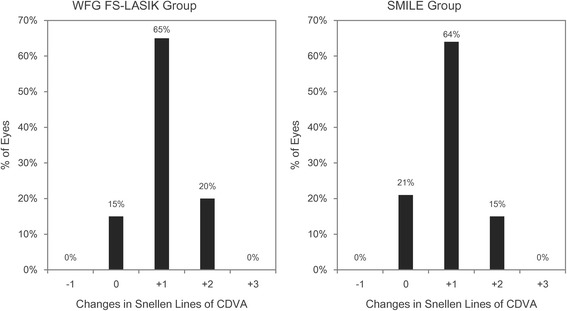
Distributions of change in Snellen lines for corrected distance visual acuity (CDVA) 3 months after wavefront-guided femtosecond LASIK (WFG FS-LASIK) and small incision lenticule extraction (SMILE)

Postoperative outcomes are shown in Table 2. There was no difference in UDVA between the two groups 3 months after surgery. Figure 1 shows no eye with a UDVA worse than 20/32 at the 3-month visit in either group. There was no difference in the percentage of eyes with a change in CDVA between the two groups and no eye lost one or more lines in CDVA (Fig. 2).

**Table 2:** Visual outcomes and ocular aberrations in the SMILE and wavefront-guided femtosecond LASIK groups 3 months after surgery

Mean ± SD (range)	WFG FS-LASIK	SMILE	*P* value^a^
Visual outcomes			
UDVA (logMAR)	−0.02 ± 0.07 (−0.18,0.15)	−0.01 ± 0.06 (−0.08,0.15)	0.732
CDVA (logMAR)	−0.04 ± 0.06 (−0.18,0.15)	−0.04 ± 0.04 (−0.08,0.00)	0.694
SE (D)	0.01 ± 0.22 (−0.50,0.50)	−0.06 ± 0.20 (−0.63,0.50)	0.128
Ocular Aberration (μm)			
Z_3_^−3^	0.072 ± 0.056 (0.001,0.275)	0.063 ± 0.045 (0.004,0.211)	0.342
Z_3_^−1^	0.116 ± 0.077 (0.027,0.304)	0.163 ± 0.093 (0.026,0.400)	0.036
Z_3_^1^	0.084 ± 0.060 (0.001,0.084)	0.090 ± 0.068 (0.002,0.250)	0.883
Z_3_^3^	0.065 ± 0.055 (0.001,0.225)	0.048 ± 0.036 (0.004,0.148)	0.399
Z_4_^0^	0.068 ± 0.053 (0.002,0.225)	0.089 ± 0.048 (0.008,0.190)	0.061
Total HOA	0.245 ± 0.094 (0.131,0.559)	0.267 ± 0.073 (0.130,0.439)	0.168

CDVA =corrected distance visual acuity (spectacles); UDVA =uncorrected distance visual acuity; SE = spherical equivalent; RMS = root mean square; ^a^ *P* values were based on Mann-Whitney test for non-normally distributed data unless otherwise specified

**Fig. 3:**
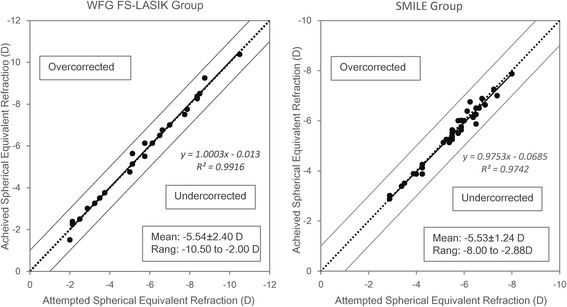
Relationship between targeted and achieved absolute spherical equivalent (SE) 3 months after surgery. The area above the diagonal dotted line indicates overcorrection and the area below the line indicates undercorrection. Regression lines and corresponding R^2^ was reported for both groups

**Fig. 4:**
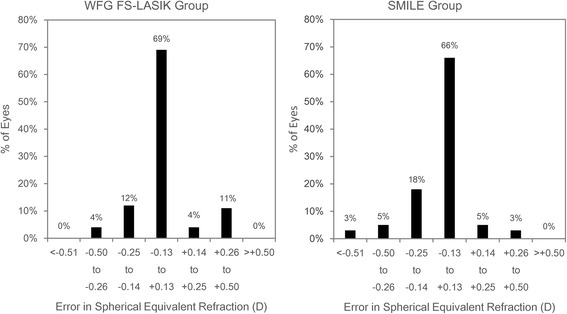
Percentages of eye of correction error in spherical equivalent (SE) (targeted correction subtracted from achieved correction) 3 months after wavefront-guided femtosecond LASIK (WFG FS-LASIK) and small incision lenticule extraction (SMILE)

Table 2 shows that there was no significant difference in the SE between the two groups 3 months after surgery. Figure 3 shows that the SE was corrected to the targeted level after both types of surgery for all eyes. There was no significant difference (Z = −0.476, *p* = −0.631) in the percentage of eyes achieving emmetropia based on a threshold of ±0.50 D between the two groups (Fig. 4).

### Changes in HOAs

**Fig. 5:**
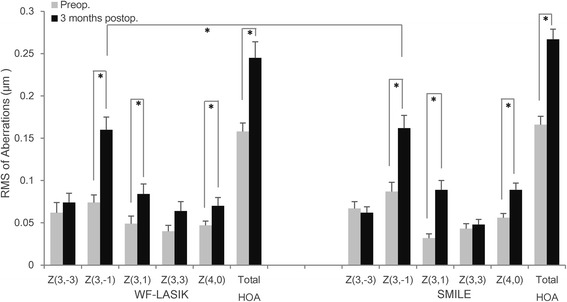
Root mean square (RMS) of total HOA and individual Zernike coefficients in wavefront-guided femtosecond LASIK (WFG FS-LASIK) and small incision lenticule extraction (SMILE) groups 3 months after surgery. Error bars indicate standard errors and asterisks indicate *p* values <0.05

Figure 5 shows that the values of coma, spherical aberration, and total HOA increased significantly in both groups 3 months after surgery as compared to the preoperative data. Table 2 shows significantly higher vertical coma values following SMILE surgery versus wavefront-guided femtosecond LASIK (Z = −2.102, *p* = 0.036); however, no significant difference was found in horizontal coma aberration. The generalized estimating equation (GEE) analysis also revealed a significant difference in vertical coma between the two groups (W = 4.901, *p* = 0.027). The trefoil was a little higher following WFG FS-LASIK in comparison to SMILE, but the difference was not significant.

### Correlation between preoperative SE and HOAs

**Fig. 6:**
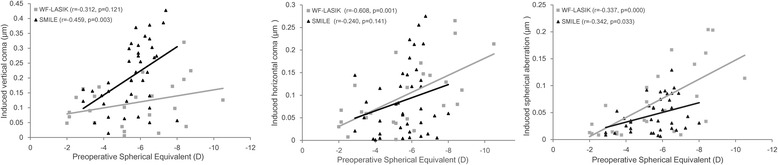
Scatter plots for preoperative spherical equivalent (SE) and changes in Zernike coefficients measured 3 months after wavefront-guided femtosecond LASIK (WFG FS-LASIK) and small incision lenticule extraction (SMILE). Regression lines and corresponding r^2^ and *p* values were reported for both groups. Left: SE and vertical coma with SMILE. Middle: SE and horizontal coma with wavefront-guided femtosecond LASIK. Right: Preoperative SE and induced spherical aberration

The left picture in Fig. 6 shows that there was a significant correlation between induced vertical coma and preoperative SE in the SMILE group but not in the WFG FS-LASIK group; there was also a significant difference in the regression slopes between the two groups (Z = 2.123, *p* = 0.038). Conversely, the middle one reveals a significant correlation between induced horizontal coma and preoperative SE in the WFG FS-LASIK group but not in the SMILE group; the regression slope, however, was not significantly different between the two groups (Z = 0.044, *p* = 0.965).

The right one shows that spherical aberration was significantly correlated to preoperative SE in both groups. There was no difference in the magnitude of the correlation for the two groups as indicated by their respective slopes (Z = 1.556, *p* = 0.125).

## Discussion

The present study investigated post-operative changes in visual outcomes and ocular aberrations in eyes undergoing SMILE and WFG FS-LASIK procedures. Our results show no significant differences in visual outcomes. The surgical efficacy, defined as the percentage of eyes achieving a UDVA of 20/20 or better, was similar for SMILE (90%) and wavefront-guided femtosecond LASIK (88%), consistent with previous reports [7, 8, 13, 17–22]. The surgical predictability was defined as the percentage of eyes corrected to within ±0.50 D of the intended correction. The present study showed that 97% of eyes after SMILE and 100% of eyes after WFG FS-LASIK met this criterion, this is also comparable to earlier studies [17–20, 22–24]. No eye lost one or more lines of CDVA postoperatively in either group. Compared to WFG FS-LASIK, the SMILE procedure achieved the same UDVA and CDVA, as well as the targeted refractive correction. Therefore, SMILE can produce safe, efficient, and predictable outcomes similar to the WFG FS-LASIK, the gold standard for correcting HOAs.

There have been several published reports investigating the change in HOAs induced by SMILE [7, 8, 12, 17, 25, 26]. The present study showed an increase in total HOAs 3 months after SMILE surgery, which is in line with these earlier findings [13, 17, 25, 26]. Consistent with previous reports, the total HOAs in our study also increased after WFG FS-LASIK [18, 21]. As we know, although the horizontal coma was mirror-symmetric, both negative and positive aberration degrade image quality, thus, the magnitudes of the aberration without regard to the sign still have the same role when evaluating optical performance. [16] Therefore, the absolute Zernike coefficients were analyzed in the present study. Finally, the present study found no significant difference in trefoil, horizontal coma, spherical aberration, and total HOA postoperatively between the two surgeries. A change in vertical coma was the single exception.

A higher vertical coma was found following SMILE as compared to WFG FS-LASIK in the present study. Li and colleagues reported that among the Zernike coefficients, vertical coma showed the greatest increase after SMILE procedure [25]. The authors of that report assumed that the increase in vertical coma after the SMILE procedure was caused by decentration of the lenticule along the vertical axis. In contrast to WFG FS-LASIK, the SMILE procedure used in the present study did not have iris registration or eye tracker, which may have resulted in less accurate centration and may explain the higher vertical coma after SMILE surgery. However, ocular aberrations were also influenced by many other factors, such as individual differences in corneal biomechanical properties and corneal wound-healing responses. SMILE is a flapless surgery with a small incision that is made at the superior aspect of the eye. Since superior-hinged flap may cause severe loss of sensation and presence of dry eye syndrome than horizontal-hinged flap in LASIK, the temporal-hinged corneal flap was made in WFG FS-LASIK in the requirement of the Ethics Committee of Tianjin Eye Hospital [14, 15]. Thus, the corneal wound-healing response at the site of the superior incision could induce an asymmetry of aberration in the vertical direction [27]. This hypothesis is also consistent with the nature of HOAs induced by LASIK, with higher horizontal coma likely induced by the flap made on the nasal side of the eye, and vertical coma by the flap at the superior location [28, 29].

A novel result was obtained in the present study. There was a significant correlation between the induced vertical coma and preoperative SE in the SMILE group, and a significant correlation between the induced horizontal coma and preoperative SE in the WFG FS-LASIK group. These findings were in line with a previous study which showed that amplitude of induced ocular coma was correlated to the diopter correction and the amount of decentration [30]. Even with the use of eye trackers, decentration may still occur because of several factors related to the patient, surgeon, and machine. Our results suggest that higher refractive corrections increases procedural time, which allows more time for loss of patient fixation and thus induces more aberration [31]. Meanwhile, the wound-healing response also differed due to two main reasons. First, there is a flap created in WFG FS-LASIK, but only a small incision in SMILE, thus SMILE might better maintain the integrity of the cornea. The second reason is the different wound-healing mechanisms related to the power of the correction [32]. It was assumed that more energy would be delivered to the cornea with WFG FS-LASIK because of the higher attempted correction, requiring more tissue to be ablated and increased exposure to the excimer laser. In contrast, energy levels were constant in SMILE and had nothing to do with the attempted correction. Additional studies are required to further evaluate these differences.

The present study also showed a positive change in spherical aberration after both SMILE and WFG FS-LASIK. The amount of induced aberration was correlated to the magnitude of the preoperative refractive error. Such an outcome could have been caused by the change in corneal asphericity after refractive surgery. The corneal shape might have changed from its natural prolate aspheric optical architecture to an oblate surface after myopic correction, which would worsen the spherical aberrations [33, 34].

A limitation of the current study is that subjective symptoms were not assessed to further evaluate visual qualities after surgery. In future research, standardized questionnaires could be utilized to find out if the visual quality was significantly impacted by the increased ocular aberration, particularly the vertical coma.

## Conclusions

In conclusion, the present study showed that both SMILE and WFG FS-LASIK were safe, efficient, and predictable procedures for myopic correction, and they produced similar changes in overall ocular aberrations. A higher vertical coma was found in SMILE than WFG FS-LASIK, and this was correlated to preoperative SE. Accurate centration during the SMILE procedure and controlling wound healing might be critical to minimize the induced coma. Further investigation is needed to verify this hypothesis.

## Abbreviations

CDVA: Corrected distance visual acuity
FL: Femtosecond laser
FLEx: Femtosecond lenticule extraction
GEE: Generalized estimating equation analyses
HOAs: Higher-order aberrations
LASIK: Laser in-situ keratomileusis WFG FS-LASIK Wavefront-guided femtosecond laser-assisted in situ keratomileusis
SE: Spherical equivalence
SMILE: Small incision lenticule extraction
UDVA: Uncorrected distance visual acuity
